# Retrospective analysis of risk factors and gaps in prevention strategies for mother-to-child HIV transmission in Rio de Janeiro, Brazil

**DOI:** 10.1186/s12889-018-6002-8

**Published:** 2018-09-10

**Authors:** Kathryn Lynn Lovero, Thais Raquelly Dourado de Oliveira, Estela Magalhães Cosme, Natália Beatriz Cabrera, Mariana Fernandes Guimarães, Juliana Gregório de Avelar, Giovanna Rodrigues Teixeira de Oliveira, Camila de Morais Salviato, Guillermo Douglass-Jaimes, Maria Leticia Santos Cruz, Esaú Custódio João, Ana Cláudia Mamede Wiering de Barros, Marcos Vinicius da Silva Pone, Ivete Martins Gomes, Lee Woodland Riley, Claudete Aparecida Araújo Cardoso

**Affiliations:** 1 Department of Psychiatry, Columbia College of Physicians and Surgeons / New York State Psychiatric Institute, 1051 Riverside Drive #24, New York, NY 10032 USA; 2 Departamento Materno-Infantil, Faculdade de Medicina, Universidade Federal Fluminense, Rua Marquês de Paraná 303, Niterói, Rio de Janeiro, 24.033-900 Brazil; 3 Division of Infectious Diseases and Vaccinology, School of Public Health, University of California Berkeley, 530E Li Ka Shing Center, Berkeley, CA 94720 USA; 4 Serviço de Doenças Infecciosas e Parasitárias, Hospital Federal dos Servidores do Estado, Rua Sacadura Cabral 178, Rio de Janeiro, Rio de Janeiro 20.221-903 Brazil; 5 Setor de Doenças Infecciosas Pediátricas, Departamento de Pediatria, Instituto Nacional de Saúde da Mulher, da Criança e do Adolescente Fernandes Figueira (FIOCRUZ), Avenida Rui Barbosa 716, Rio de Janeiro, Rio de Janeiro 22.250-020 Brazil; 6 Setor de Doenças Sexualmente Transmissíveis, Hospital Geral de Nova Iguaçu, Avenida Henrique Duque Estrada Mayer 953, Nova Iguaçu, Rio de Janeiro, 26.050-210 Brazil

**Keywords:** Vertical transmission, PMTCT programme evaluation, Missed opportunities, Prevention gaps, Brazil, HIV

## Abstract

**Background:**

Despite great progress made in methods to prevent mother-to-child transmission of HIV (MTCT), delivery and uptake of these measures remains a challenge in many countries. Although the Brazilian Ministry of Health aimed to eliminate MTCT by 2015, infection still occured in 15–24% of infants born to HIV-infected mothers. We sought to identify remaining factors that constrain MTCT elimination.

**Methods:**

We conducted a retrospective, matched case-control study by reviewing hospital charts of infants born to HIV-infected mothers between 1997 and 2014 at three MTCT reference hospitals in the Rio de Janeiro metropolitan area. Cases were defined as HIV-exposed children with two positive HIV tests before 18 months of age; controls were defined as HIV-exposed children with two negative HIV tests before 18 months of age. We performed bivariate and MTCT cascade analyses to identify risk factors for MTCT and gaps in prevention services.

**Results:**

We included 435 infants and their mothers (145 cases, 290 controls). Bivariate analyses of MTCT preventative care (PMTCT) indicated that cases were less likely to complete all individual measures in the antenatal, delivery, and postnatal period (*p* < 0.05). Assessing completion of the PMTCT cascade, the sequential steps of PMTCT interventions, we found inadequate retention in care among both cases and controls, and cases were significantly less likely than controls to continue receiving care throughout the cascade (*p* < 0.05). Motives for incompletion of PMTCT measures included infrastructural issues, such as HIV test results not being returned, but were most often due to lack of care-seeking. Over the course of the study period, PMTCT completion improved, although it remained below the 95% target for antenatal care, HIV testing, and antenatal ART set by the WHO. Adding concern, evaluation of co-infections indicated that case infants were also more likely to have congenital syphilis (OR: 4.29; 95% CI: 1.66 to 11.11).

**Conclusions:**

While PMTCT coverage has improved over the years, completion of services remains insufficient. Along with interventions to promote care-seeking behaviour, increased infrastructural support for PMTCT services is needed to meet the HIV MTCT elimination goal in Brazil as well as address rising national rates of congenital syphilis.

## Background

Without prophylactic treatment, mother-to-child transmission (MTCT) occurs in approximately 20–45% of infants born to HIV-infected women [1]. However, with current measures to prevent MTCT (PMTCT), the frequency can be reduced to < 2% in non-breastfeeding populations [2]. Despite having an internationally recognized approach to the prevention and treatment of HIV, Brazil has not yet reached its goal of elimination of MTCT [3]. In 2011, the Brazilian Ministry of Health (BMoH) launched the “Stork Network” (“Rede Cegonha”) [4] in support of the UNAIDS goal to eliminate MTCT by 2015 [5], strengthening efforts toward early testing and antiretroviral (ARV) administration within the public health system. However, by 2015, the frequency of MTCT in Brazil was estimated to still be between 15 and 24% [6].

Per Brazilian national guidelines, the series of interventions used to identify and treat HIV-infected pregnant women and their infants, known as the PMTCT cascade [7], begin with antenatal care, where a pregnant woman is tested for HIV and administered antiretroviral therapy (ART) if found to be infected. Starting in 1996, antenatal HIV-infected women were offered zidovudine (AZT); in 2007, all HIV-infected women with CD4 cell count of < 200 cells/mL and/or plasma viral load of > 1000 copies/mL were offered triple ARV prophylaxis; since 2010, all HIV-infected women have been offered triple ARV prophylaxis in the antenatal period [8]. During delivery, HIV-infected pregnant women receive intravenous infusion of AZT, and infants are delivered by Caesarean section if maternal viral load is > 1000 copies/mL or unknown. Finally, during the postnatal period, HIV-exposed infants are treated with oral ARVs and fed a formula-only diet when possible. Supporting the PMTCT cascade, Brazil has offered free ART nationwide since 1996 [9], and has provided free formula for HIV-exposed infants since 2002 [8].

Because of the failure of current prevention efforts to reduce MTCT to targeted levels, in 2014 the BMoH called for increased research into vertical transmission to identify treatment gaps and underserved populations [10]. In the present study, we conducted a retrospective analysis to evaluate completion of the PMTCT cascade and determine risk factors for MTCT at three PMTCT reference hospitals in Greater Rio de Janeiro, the second most populous metropolitan area in Brazil.

## Methods

### Study setting and population

This retrospective case-control study was carried out at three PMTCT reference hospitals in the Rio de Janeiro metropolitan area. The Instituto Nacional de Saúde da Mulher, da Criança e do Adolescente Fernandes Figueira (IFF), located in the south zone of Rio de Janeiro, and the Hospital Federal dos Servidores do Estado (HFSE), located in the city centre, are tertiary hospitals that serve as the PMTCT reference centres for 58% of primary care units in Rio de Janeiro [11]. The Hospital Geral de Nova Iguaçu (HGNI) is located in a region of suburbs north of Rio de Janeiro called the Baixada Fluminense, and an estimated 12% of HIV-infected women in the state of Rio de Janeiro receive care at this hospital [12].

We reviewed hospital records to identify infants born to HIV-infected mothers between 1997, when ARVs became universally available in Brazil for PMTCT, and 2014. Cases were defined as HIV-exposed children with two detectable HIV viral load tests; controls were defined as HIV-exposed children with two undetectable HIV viral load tests, at least one of which done after 4 months of age. For each included case, two time- and study site-matched controls—the last HIV-exposed, uninfected infant born before the case and the next HIV-exposed, uninfected infant born after the case at the same hospital—were selected to control for evolving PMTCT interventions provided over the study period. HIV-exposed children who were diagnosed as HIV-infected or uninfected after 18 months of age were excluded from the study, as their medical records do not regularly contain any information on the mothers’ sociodemographic data nor antenatal and postnatal care. Second-born twins were also excluded, as they have the same exposures but reduced risk of MTCT compared to first borns [13]. Additionally, mothers who were HIV-test negative during delivery were excluded, as MTCT risk factors evaluated during the antenatal period would not apply.

### Measures and data collection

Data were collected from mother and child medical records on a standardized intake form, based on the 2014 Protocol for Investigation of Vertical Transmission from the BMoH [10]. The intake form included sociodemographic data of the mother as well as standard information on antenatal care, birth, and postnatal care. All sociodemographic data reflect participant-reported information except for participant’s race, which in Brazilian medical records is defined by the attending medical professional.

### Data analysis

All data were analysed by Stata IC14 (Statacorp, College Station, USA). We compared characteristics of matched cases (HIV-infected infants and their mothers) and controls (HIV-uninfected infants and their mothers) with conditional logistic regression, which fits models for 1:x matched case-control data (1:2 in the present study). Indicator variables were created from categorical variables for regression analyses, and reference categories are indicated as 1 in analysis tables. To evaluate residence in informal settlements, we geocoded participant addresses with Google Geocoding API v.3 (https://developers.google.com/maps/documentation/geocoding/start). Participants whose addresses were incomplete (*n* = 60), could not be geocoded (*n* = 18), or were homeless (*n* = 2) were excluded from analysis. Remaining addresses were mapped to census tracts (http://www.censo2010.ibge.gov.br) for federally recognized informal settlements known as “aglomerados subnormais” (AGSN) [14] with ArcGIS 10.4.1 (ESRI, Redlands, CA, USA).

## Results

### Study population

**Fig. 1:**
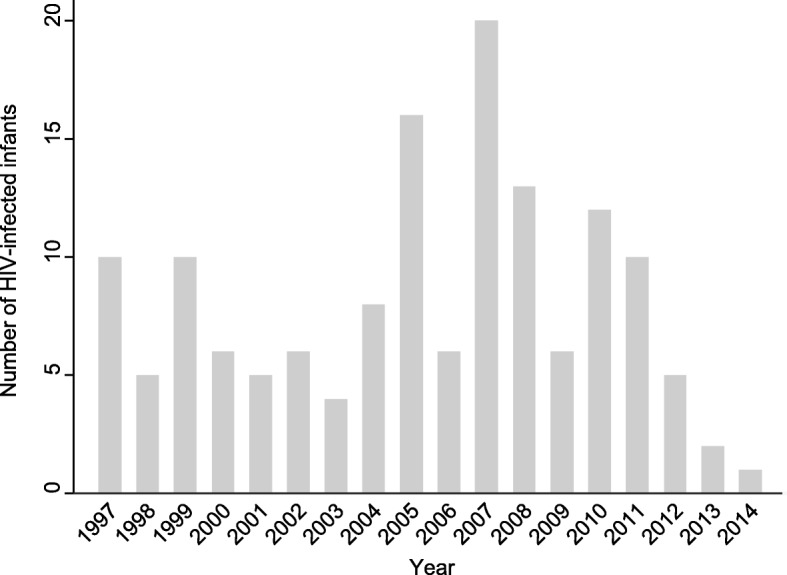
Number of cases included at study sites for each year of study period

Between 1997 and 2014, 266 MTCT cases were documented at the three research sites. Of these, 121 were excluded for the following reasons: 35 (28.9%) medical records not located, 2 (1.7%) second twins, 8 (6.6%) HIV-uninfected mothers at delivery, and 76 (62.8%) infants diagnosed after 18 months of age. For the 145 cases included in our study, two time-matched controls were chosen at the cases’ respective hospitals (IFF: *n* = 55 (37.9%) cases, *n* = 110 (37.9%) controls; HFSE: *n* = 38 (26.2%) cases, *n* = 76 (26.2%) controls; HGNI: *n* = 52 (35.9%) cases, *n* = 104 (35.9%) controls). Analysis of cases over the study period showed that after an initial decline, an increase in cases were reported between 2005 and 2008, but decreased again so that by 2014 only one case was identified at our study sites (Fig. 1).

### Sociodemographic characteristics

The median age at delivery was 26 years (IQR: 22 to 32) for case and 27 (IQR: 22 to 31) years for control mothers. Between time of HIV diagnosis and the pregnancy under evaluation, case mothers were more likely than control mothers to have not given birth to another child (OR: 1.97; 95% CI 0.99 to 3.90). No significant differences were observed in race, marital status, education level, employment, drug use, and AGSN residence (Table 1).

**Table 1:** Sociodemographic characteristics of case and control mothers

	Controls (*n* = 290) N (%)	Cases (*n* = 145) N (%)	OR (95% CI)	*p* ^a^
Median Age, Years (Interquartile Range)	26 (22–32)^b^	27 (22–31)^c^	1.01 (0.97–1.05)	0.64
Race				
White	94 (32.4)	28 (19.3)	1	
Mixed Race	97 (33.5)	40 (27.6)	1.72 (0.90–3.29)	0.10
Black	53 (18.3)	30 (20.7)	1.30 (0.73–2.32)	0.37
No information	46 (15.9)	47 (32.4)	4.21 (2.17–8.13)	< 0.000
Marital Status				
Single	67 (23.1)	40 (27.6)	1	
Married	119 (41.0)	55 (37.9)	0.77 (0.47–1.29)	0.32
No information	104 (35.9)	50 (34.5)	0.80 (0.46–1.39)	0.43
Education Level				
None	7 (2.4)	3 (2.1)	1	
Some or all primary school	167 (57.6)	72 (49.7)	1.42 (0.22–9.36)	0.71
Some or all secondary school	61 (21.0)	18 (12.4)	1.07 (0.15–7.60)	0.94
Some or all college	5 (1.7)	1 (0.7)	0.89 (0.05–16.10)	0.94
No information	50 (17.2)	51 (35.2)	4.67 (0.65–33.60)	0.13
Employment				
Unemployed/Home Maker	126 (43.5)	52 (35.9)	1	
Employed	69 (23.8)	21 (14.5)	0.80 (0.43–1.47)	0.47
Student	8 (2.8)	2 (1.4)	0.58 (0.12–2.81)	0.50
No information	87 (30.0)	70 (48.3)	2.29 (1.36–3.83)	0.002
Drug Use				
Yes	44 (15.2)	22 (15.2)	1	
No	102 (35.2)	38 (26.2)	0.77 (0.41–1.46)	0.43
No information	144 (49.7)	85 (58.6)	1.48 (0.75–2.95)	0.26
Residence				
AGSN	49 (16.9)	14 (9.7)	1	
Non-AGSN	207 (71.4)	87 (60.0)	1.49 (0.78–2.85)	0.23
No information	34 (11.7)	44 (30.3)	5.68 (2.50–12.88)	< 0.000
Other Child Since Diagnosis				
Yes	53 (18.3)	14 (9.7)	1	
No	164 (56.6)	83 (57.2)	1.97 (0.99–3.90)	0.05
No information	73 (25.2)	48 (33.1)	2.74 (1.3105.71)	0.007

^a^Conditional logistic regression; ^b^*n* = 252; ^c^*n* = 105

### PMTCT measures in cases and controls

In bivariate analysis of PMTCT measures, we found case mothers and infants were more likely to have not completed each preventative step during antenatal care, delivery, and postnatal care (Table 2). Case mothers were more likely to have had no antenatal care (OR: 3.10, 95% CI: 1.68 to 5.70), and were more likely to not have been diagnosed as HIV-infected until delivery (OR: 3.68, 95% CI: 1.90 to 7.14) or after the birth of their child (OR: 11.21, 95% CI: 5.27 to 23.36. Of women who completed at least one antenatal visit, there was no significant difference in gestational age at which case and control mothers entered antenatal care. However, case mothers were more likely to not antenatal care at an HIV reference centre (OR: 19.78, 95% CI: 5.65 to 69.24), where it is protocol that all HIV-infected mothers be referred after a positive diagnosis [15]. Additionally, case mothers were more likely to not initiate or complete antenatal ART (aART) (OR: 19.41, 95% CI: 8.72 to 43.21). Evaluating only mothers who completed aART, there was no significant difference in ART regimen. During delivery, case mothers were more likely to not be administered intravenous AZT (OR: 4.89, 95% CI: 2.91 to 8.24) and to not deliver by Caesarean section (OR: 1.73, 95% CI: 1.11 to 2.71). No significant difference was found in the gestational age at birth, nor in membrane rupture times less than 4 h long (data not shown). In the postnatal period, case infants were more likely to not use oral ARVs (OR: 12.10, 95% CI: 5.79 to 25.27) and to be breastfed (OR: 5.44, 95% CI: 2.84 to 10.40).

**Table 2:** PMTCT measures in case and control mothers

	Controls (*n* = 290) N (%)	Cases (*n* = 145) N (%)	OR (95% CI)	*p* ^a^
Antenatal Care				
Yes	213 (73.5)	60 (41.2)	1	
No	32 (11.0)	29 (20.0)	3.10 (1.68–5.70)	< 0.000
No information	45 (15.5)	68 (38.6)	5.62 (3.11–10.13)	< 0.000
Time of Diagnosis				
Before/during antenatal	192 (66.2)	41 (28.3)	1	
During delivery	56 (19.3)	36 (24.8)	3.68 (1.90–7.14)	< 0.000
After birth	14 (4.8)	36 (24.8)	11.21 (5.27–23.36)	< 0.000
No information	28 (9.7)	32 (22.1)	6.01 (2.96–12.19)	< 0.000
Gestational Age at First Visit	18.8 (0.9)^b^	22.3 (2.4)^c^	1.07 (0.99–1.15)	0.11
Antenatal Location				
Some or all in reference centre	157 (60.9)	14 (12.1)	1	
No reference centre	56 (21.7)	46 (39.7)	19.78 (5.65–69.24)	< 0.000
No information	45 (17.4)	56 (48.3)	37.47 (10.73–130.84)	< 0.000
Antenatal ART				
Yes	169 (58.3)	16 (11.0)	1	
No	95 (32.8)	101 (69.7)	19.41 (8.72–43.21)	< 0.000
No information	26 (9.0)	28 (19.3)	14.91 (5.71–38.93)	< 0.000
Antenatal ART Regimen				
Triple ARV	71 (42.0)	8 (50.0)	1	
AZT only	98 (58.0)	8 (50.0)	1.38 (0.43–4.43)	0.54
Intravenous AZT during Delivery				
Yes	190 (65.5)	41 (28.3)	1	
No	55 (19.0)	62 (42.8)	4.89 (2.91–8.24)	< 0.000
No information	45 (15.5)	42 (29.0)	4.31 (2.38–7.80)	< 0.000
Type of Birth				
Caesarean	174(60.0)	59 (40.7)	1	
Vaginal	94 (32.4)	57 (39.3)	1.73 (1.11–2.71)	0.02
No information	22 (7.6)	29 (20.0)	3.74 (1.98–7.09)	< 0.000
ART in Infant				
Yes	250 (86.2)	65 (44.8)	1	
No	12 (4.1)	42 (29.0)	12.10 (5.79–25.27)	< 0.000
No information	28 (9.7)	38 (26.2)	8.60 (4.04–18.26)	< 0.000
Formula Only				
Yes	243 (83.8)	74 (51.0)	1	
No	15 (5.2)	31 (21.4)	5.44 (2.84–10.40)	< 0.000
No information	32 (11.0)	40 (27.6)	4.87 (2.60–9.15)	< 0.000

^a^Conditional logistic regression; ^b^*n* = 101; ^c^*n* = 19

### Completion of PMTCT cascade

**Fig. 2:**
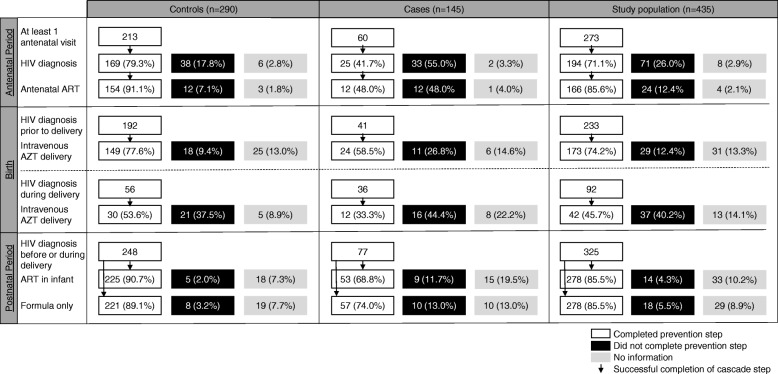
Completion of PMTCT cascade. Number and percentage, in parentheses, of study participants who completed, did not complete, or lacked information on each preventative measure they were known to be eligible to receive. Arrows indicate steps in completion of the PMTCT cascade.

While the analysis in Table 2 provides an overall assessment of the completion of each PMTCT measure, because these measures are dependent upon one another, it may be that the differences seen between cases and controls throughout the PMTCT cascade are driven by incompletion of earlier steps by cases. For example, a woman who was not diagnosed as HIV-infected before or during delivery would not have been eligible to receive aART or intravenous AZT in delivery. To determine if differences in PMTCT completion between cases and controls persisted independent of prior gaps in care, we next analysed completion of the PMTCT cascade only among participants eligible for each step (Fig. 2).

Of women who attended at least one antenatal visit (*n* = 213 controls, *n* = 60 cases), just 25 case (41.7%) compared to 169 control (79.3%) mothers were diagnosed as HIV-infected (OR: 0.25, 95% CI: 0.10 to 0.65). Of these women who were known to be HIV-infected in the antenatal period, only 12 (48%) case compared to 154 (91.1%) control mothers completed aART (OR: 0.08, 95% CI: 0.03 to 0.24). However, when we evaluated only women known to be HIV-infected who attended any antenatal care at a PMTCT reference centre, per Brazilian protocol for HIV-infected expectant mothers, there was no significant differences in the portion of case (*n* = 11, 100%) compared to control (*n* = 148, 97.3%) mothers who completed aART. Additionally, considering only mothers who had been diagnosed but had no or unknown antenatal care, only 3 cases (15%) compared to 12 controls (50%) completed aART (OR: 0.18, 95% CI: 0.03 to 0.88).

Evaluating completion of the PMTCT cascade during delivery, we first assessed all mothers who had been diagnosed before or during the antenatal period, regardless of whether their records showed evidence of any antenatal care (*n* = 192 controls, *n* = 41 cases), as this is not necessary for completion of cascade steps we evaluated during delivery. Of these mothers, just 24 (58.5%) cases compared to 149 (77.6%) controls received intravenous AZT during delivery (OR: 0.17, 95% CI: 0.03 to 0.80). However, of mothers diagnosed as HIV-infected during delivery (*n* = 56 controls, *n* = 36 cases), only 12 (33.3%) cases and 30 (53.6%) control mothers received intravenous AZT (OR: 0.27, 95% CI: 0.06 to 1.27).

Assessing interventions in the postnatal period, 248 control and 77 case infants were born to women who had been diagnosed before or during delivery. Of these infants, only 53 (68.8%) cases compared to 225 (90.7%) controls were treated with oral ARVs (OR: 0.14, 95% CI: 0.03 to 0.67). Moreover, just 57 (74%) case compared to 221 (89.1%) control infants were fed a formula-only diet (OR: 0.28, 95% CI: 0.09 to 0.83).

### Progress towards the elimination of MTCT

**Fig. 3:**
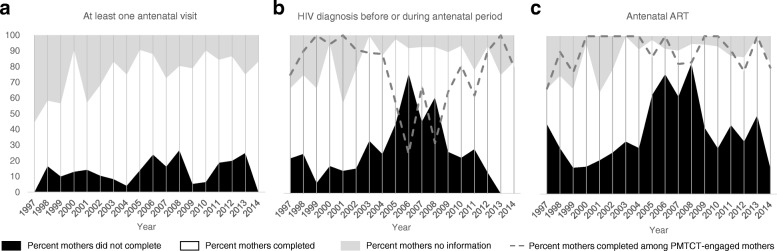
Progress toward elimination of MTCT over study period. Shaded regions indicate the percentage of study participants who did not complete (black), completed (white), and did not have information (grey) on antenatal care (**a**), HIV diagnosis (**b**), and antenatal ART (**c**). Dashed lines indicate percentage of mothers who attended antenatal care that received HIV diagnosis (**b**), and percentage of mothers who were diagnosed that completed antenatal ART (**c**)

In its 2017 guide to elimination of MTCT (EMTCT), the WHO laid out indicators on the path to EMTCT, including 1) at least one antenatal visit, 2) coverage of HIV testing for pregnant women, and 3) aART coverage in HIV-infected pregnant women [16]. To determine progress toward these goals, we assessed antenatal care attendance, antenatal diagnosis, and aART completion in all mothers across the study period (Fig. 3). Engagement in antenatal care improved over the years, though only 83.3% (*n* = 5) of mothers were known to have attended at least one visit in 2014 (Fig. 3a). HIV diagnosis before or during the antenatal period decreased between 2005 and 2010, but increased again in the final years of the study period, such that 83.3% (*n* = 5) of mothers were diagnosed in 2014. To evaluate completion of the PMTCT cascade, we also assessed HIV diagnosis only in women who attended any antenatal care, and found it increased only slightly over time, with just 80% (*n* = 4) of mothers diagnosed in 2014 (Fig. 3b). Completion of aART increased over the study period, although only 66.7% (*n* = 4) of women were shown to have completed aART in 2014. Again, evaluating only mothers engaged in the PMTCT cascade (those who attended any antenatal care and were diagnosed as HIV-infected), we found that aART completion among these women remained fairly high throughout the study period, although still just 80% (*n* = 4) of mothers completed aART in 2014 (Fig. 3c).

### Motives for not completing PMTCT steps

Our analysis of completion of the PMTCT cascade (Figs. 2 and 3) demonstrated that a large portion of mothers who entered antenatal care did not complete all antenatal steps. To better understand the contribution of lack of care-seeking, care abandonment, and failure to provide services to gaps in PMTCT coverage, we assessed the motives for which mothers were not diagnosed in the antenatal period and did not initiate or complete aART. Motives were classified based on the BMoH protocol for investigation into vertical transmission of HIV [10]. Of the 70 control mothers who were not diagnosed before or during the antenatal period, 18 (25.7%) did not attend any antenatal care, 3 (4.3%) did not have an HIV test ordered, 6 (8.6%) did not receive test results, 1 (1.4%) did not have HIV testing available at the clinic, 2 (2.9%) abandoned antenatal care, and 40 (57.1%) had unknown motives. Of the 72 case mothers not diagnosed before or during the antenatal period, 14 (19.4%) did not attend any antenatal care, 3 (4.2%) did not have an HIV test ordered, 13 (18.1%) did not receive test results, 2 (2.8%) did not have HIV testing available at the clinic, and 40 (55.6%) had unknown motives. We next evaluated motives for incomplete aART in mothers who had been diagnosed before or during the antenatal period. Of the 25 control mothers who were diagnosed as HIV infected and did not complete aART, 13 (52%) did not attend any antenatal care, 3 (12%) were not offered aART, 1 (4%) refused/abandoned treatment, 2 (8%) initiated antenatal care too late in pregnancy to begin ART, and no information on motive was available for 6 (24%). Of the 29 case mothers who were diagnosed but did not complete aART, 11 (37.9%) did not attend any antenatal care, 1 (3.4%) was not offered aART, 3 (10.3%) refused/abandoned treatment, 2 (6.9%) initiated antenatal care too late in pregnancy to begin ART, and no information on motive was available for 12 (41.4%).

### Coinfections associated with MTCT

In comparing frequencies of other congenital infections, we found that 21 (14.5%) case but only 10 (3.5%) control infants were diagnosed to have syphilis (OR: 4.29; 95% CI: 1.66 to 11.11). There was no significant difference in Hepatitis C, Hepatitis B, CMV, or toxoplasmosis infection of case and controls infants.

## Discussion

Despite innovative and aggressive public health efforts made in the prevention and treatment of HIV infection [17], Brazil has still not achieved its stated goal for 2015 of eliminating MTCT [10]. Here, we found that cases of MTCT were significantly less likely to complete all PMTCT measures in the antenatal, perinatal, and postnatal periods. Evaluating the PMTCT cascade, we identified inadequate retention in care throughout the cascade for both cases and controls, although cases were significantly less likely than controls to continue receiving care throughout the cascade. Importantly, temporal analysis of antenatal PMTCT steps showed that, although PMTCT completion has increased modestly over the study period, the study sites are still not meeting the 95% coverage goal for antenatal care, HIV testing, and aART set by the WHO.

Research across various regions of Brazil has shown that lack of antenatal care and late diagnosis underlie substantial gaps in the PMTCT cascade [8, 12, 18–20]. In our cohort, while 19% of case and 29% of control mothers were not diagnosed prior to birth because they had not attended any or abandoned antenatal care, 25% of case and 14% of control mothers did not receive their diagnosis due to a failure in antenatal services—tests not being available, not ordered, or results not reported. Moreover, 55% of case and 64% of controls mothers who were diagnosed as HIV-infected did not complete aART because of failure to seek antenatal care or refusal/abandonment of treatment, although 12% of controls and 3% of cases were not offered aART. Infrastructural issues—including long delays in scheduling antenatal visits [21, 22], lack of HIV testing services delays in reporting results [22, 23], and insufficient training of healthcare workers [21, 23]—all have been shown to contribute to decreased PMTCT initiation and completion in Brazil. Therefore, in addition to promoting care-seeking behaviour among Brazilian mothers, it is also important to address issues in availability of antenatal services and training of workers.

In addition to missed opportunities owing to lack of antenatal care and late HIV diagnosis, we identified gaps in treatment for women who were aware of their HIV status throughout the PMTCT care cascade. In the antenatal period, only 86% of the mothers who attended antenatal care and received their HIV diagnosis completed aART. Previous research in other regions of Brazil has shown similar percentages of pregnant women not completing aART. In the northern state of Amazonas, aART completion increased only 10% between 1999 and 2011, with 20% of women not completing ART in 2011 [8]. A similar situation was shown in the southern state of Rio Grande do Sul, where aART use grew 13% between 1998 and 2011, but over 25% of women still did not complete aART between 2005 and 2011 [24]. Moreover, a study conducted in the southern state of Paraná found that 16% of women did not complete aART between 2007 and 2012 [25]. However, UNAIDS reported over 95% of all pregnant women to be receiving aART in Brazil in 2015 [26]. It is possible that the aART gap closed significantly in one year, or that these and our studies are not representative of Brazil as a whole, but more likely that this difference reflects a disparity in the number of women receiving versus completing aART in Brazil. Supporting this hypothesis, 10% of the case and 4% of controls mothers in our study were prescribed aART but abandoned treatment. Notably, 100% of case and 97% of control mothers who attended antenatal care at a reference centre completed aART, indicating that a focus on improving the connection between general and PMTCT reference antenatal services may help close the gap in aART completion.

Other significant missed opportunities for PMTCT were observed in delivery and postnatal care of our study cohort. Caesarean section is recommended in women with unknown or viral loads above 1000 copies/mL [15]. Because the medical records lacked data on maternal viral load, we could not precisely define the percentage of women who did not undergo Caesarean section in cases where protocol would have called for it. However, knowing that 70% of case mothers did not complete aART, and only 41% delivered by Caesarean section, it is clear that a large portion of case mothers in our study, whose HIV infection was not managed in the antenatal period, did not undergo Caesarean section. Additionally, only 66% of mothers who were diagnosed before or during delivery received intravenous AZT during delivery, and just 86% of infants born to mothers who had been diagnosed as HIV-infected used oral ARVs and were exclusively formula fed, supporting the need for increased efforts to promote completion of the PMTCT cascade beyond antenatal care.

Another important finding in our study is that the frequency of syphilis was significantly higher in case infants. In Brazil, reports of congenital syphilis grew by over 300% between 2005 and 2015, and 44% of syphilis-infected pregnant women were not diagnosed until after delivery [27]. Dual elimination of mother-to-child transmission of HIV and syphilis was recently recognized by the WHO as a global health priority [28]. Occurrence of syphilis in the case women in our study most likely reflects absence of antenatal screening, and efforts to improve uptake and retention in antenatal HIV services can also support reduction in MTCT of syphilis.

Although there are still gaps in preventative treatment, the population not receiving care in Brazil is small. However, especially considering rising HIV rates in Brazil over the last decade [29], to achieve EMTCT this population must be identified and targeted. Our analysis of sociodemographic factors did not reveal any significant differences between cases and controls. Owing to our use of retrospective data collection, this may be due to high proportions of missing sociodemographic data. Previous research has shown that psychosocial factors—including stigma, lack of social support, and distrust or misunderstanding of antenatal care [21]--contribute to decreased PMTCT initiation and completion in Brazil. Thus, prospective studies on sociodemographic indicators of MTCT risk as well as efforts to address psychosocial barriers must be carried out together to achieve elimination goals.

Due to the retrospective nature of this study, we were limited to collecting data commonly found in medical records and thus were not able to evaluate maternal viral load and other socioeconomic indicators. Additionally, we were unable to obtain complete data from medical charts of many of the study participants, due to missing information. Interestingly, cases were more likely to have missing information, which suggests that they were less engaged in care throughout the PMTCT cascade. While our use of matched controls allowed us to control for changes in provision of services (e.g. government-provided formula in 2002) and improvements in PMTCT regimens (e.g. triple ARV introduction in 2007) when assessing risk factors for MTCT, it limited our analysis in that we were not able to evaluate potential differences between PMTCT sites.

## Conclusions

Although PMTCT coverage improved over the course of the study period, to succeed in eliminating MTCT of HIV in Brazil, it is critical that efforts be made to increase uptake and retention in PMTCT services to promote early diagnosis and completion of all steps in the PMTCT cascade. Specifically, identification of socioeconomic and psychosocial barriers to accessing care as well as improvements in PMTCT infrastructure are required. The impact of improvements in antenatal care has significance beyond reductions in HIV transmission, as increased engagement in antenatal care is also critical to addressing rising national rates of congenital syphilis.

## Abbreviations

aART: antenatal ART
AGSN: Aglomerados subnormais (Brazilian census-defined slums)
aOR: Adjusted odds ratio
ART: Antiretroviral therapy
ARV: Antiretroviral
AZT: Azidothymidine/zidovudine
BMoH: Brazilian Ministry of Health
CI: Confidence interval
CMV: Cytomegalovirus
HFSE: Hospital Federal dos Servidores do Estado (HFSE)
HGNI: Hospital Geral de Nova Iguaçu
IFF: Instituto Nacional de Saúde da Mulher, da Criança e do Adolescente Fernandes Figueira
IQR: Interquartile range
MTCT: Mother-to-child transmission
OR: Odds ratio
PMTCT: Prevention of mother-to-child transmission
